# Prevalence and determinants of substance use among university students in Saudi Arabia: a nationwide online cross-sectional study

**DOI:** 10.3389/fpubh.2026.1841671

**Published:** 2026-06-01

**Authors:** Najim Z. Alshahrani, Mohamed Baklola, Mohamed Terra, Ahmed K. Shukri, Ghada Elkhawaga, Mohannad A. Alzain

**Affiliations:** 1 Department of Family and Community Medicine, Faculty of Medicine, University of Jeddah, Jeddah, Saudi Arabia; 2 Faculty of Medicine, Mansoura University, Mansoura, Egypt; 3 Department of Family and Community Medicine, Faculty of Medicine, Jouf University, Jouf, Saudi Arabia; 4 Department of Family Medicine, Faculty of Medicine, King Abdulaziz University, Jeddah, Saudi Arabia; 5 Department of Family Medicine, King Abdulaziz University Hospital, King Abdulaziz University, Jeddah, Saudi Arabia; 6 Family Medicine and Chronic Diseases Research Unit, King Fahd Medical Research Center, King Abdulaziz University, Jeddah, Saudi Arabia

**Keywords:** ASSIST, Saudi Arabia, substance use, tobacco use, university students

## Abstract

**Background:**

Substance use among university students is a growing public health concern, particularly in sociocultural contexts where reporting may be constrained by sociocultural stigma. National data on substance use patterns and determinants among university students in Saudi Arabia remain limited. This study aimed to determine the prevalence and determinants of substance use among a multi-regional sample of university students in Saudi Arabia.

**Methods:**

A nationwide online cross-sectional study using convenience sampling was conducted among university students in Saudi Arabia between 1 November and 30 December 2025. Data were collected using an anonymous online questionnaire adapted from the Arabic version of the World Health Organization’s Alcohol, Smoking, and Substance Involvement Screening Test (ASSIST v3.0), restricted to lifetime substance use items. A total of 744 students from more than ten universities across all regions of the country participated. Descriptive analyses, bivariate analyses, and multivariable logistic regression were conducted using R version 4.3.3 to identify factors associated with lifetime tobacco use. Statistical significance was set at a two-sided *p* value of <0.05.

**Results:**

The lifetime prevalence of any substance use, including tobacco, was 45%. Tobacco was the most commonly reported substance (30.0%). Excluding tobacco, 15% of participants reported lifetime use of at least one non-tobacco substance (participants could report more than one substance), most frequently sedatives and sleeping pills (14.0%) and cannabis (9.7%). Marked gender differences were observed among substance users, with males demonstrating higher use of tobacco, cannabis, alcohol, and amphetamine-type stimulants, while sedative use was more prevalent among females. In multivariable analysis, older age, male gender, enrollment in non-medical faculties, and peer pressure were independently associated with higher odds of lifetime tobacco use, whereas higher academic performance was associated with lower odds.

**Conclusion:**

Substance use is relatively common among university students in Saudi Arabia and exhibits distinct gender-specific patterns. These findings should be interpreted in light of the use of lifetime measures, which limit assessment of current use and severity. Targeted, culturally sensitive prevention strategies addressing tobacco use, academic environments, and peer influence are needed.

## 1 Introduction

Substance use among university students represents a major global public health concern, due to its association with adverse physical, psychological, and social outcomes, as well as its potential to impair academic performance and long-term productivity (1). Young adulthood is a critical developmental period during which experimentation with psychoactive substances often begins, and behaviors established at this stage may persist into later life, increasing the risk of substance use disorders and chronic health conditions (2). Globally, substance use contributes substantially to morbidity and mortality, accounting for a significant proportion of disability-adjusted life years (DALYs) and preventable deaths, particularly among young adults (3).

Tobacco remains the most commonly used substance globally, with a daily smoking prevalence of 15.2%, followed by heavy episodic alcohol (18.3%), cannabis (3.8%), opioids (0.37%), amphetamines (0.77%), and cocaine (0.35%). Cannabis is the most seized illicit drug, used by 147 million annually (2.5% of the world population aged 15–64), yet remains far less prevalent than tobacco and alcohol (4, 5). In many Western countries, alcohol use predominates among university students, whereas in more conservative settings, tobacco and prescription medications are more commonly reported (6–8). These contextual differences underscore the importance of region-specific data to inform prevention strategies.

In Saudi Arabia, substance use is shaped by strict legal restrictions, religious norms, and cultural values that prohibit alcohol and illicit drug use (9). Despite these deterrents, tobacco use remains prevalent, particularly among adolescents and young adults, and has been identified as one of the most commonly abused substances nationwide. Emerging evidence also suggests increasing misuse of prescription medications, such as sedatives and stimulants, especially among students in health-related disciplines (10). These substances are often perceived as less harmful or more socially acceptable, despite their well-documented risks.

University students represent a particularly important population for substance use research. Academic pressure, transition to independent living, exposure to new social networks, and mental health challenges may collectively increase vulnerability to substance use during this period (11). Medical and health sciences students face additional stressors, including demanding curricula and high expectations, which may further contribute to maladaptive coping behaviors. Importantly, substance use among future healthcare professionals carries implications beyond individual health, as it may influence professional behavior, clinical judgment, and attitudes toward patient counseling (12).

Although several studies have examined substance use among university students in Saudi Arabia, most have focused on single institutions, specific regions, or limited substance categories or the general population. National-level data examining a broad range of substances and associated academic and psychosocial factors remain scarce among university students. A clearer understanding of current prevalence patterns and determinants is therefore essential to inform targeted prevention programs, strengthen student support services, and guide policy development within higher education institutions. This study aimed to determine the prevalence and determinants of substance use among a multi-regional sample of university students in Saudi Arabia. This cross-sectional study has been reported in line with the STROCSS 2025 guidelines (13).

## 2 Methods

### 2.1 Study design, setting, and period

A nationwide online cross-sectional study with an analytic component was conducted among a multi-regional sample of university students in Saudi Arabia. Data collection was carried out over a two-month period from 1 November 2025 to 30 December 2025 using an anonymous, self-administered online questionnaire. The study targeted undergraduate students enrolled in public and private universities across all five geographical regions of the country, allowing participation from multiple regions and a range of academic disciplines.

### 2.2 Sampling strategy and data collection

A convenience sampling approach was employed. The survey was administered electronically using Google Forms and disseminated broadly through official and informal university-affiliated social media platforms, including WhatsApp and Telegram groups. Official platforms included university-managed student groups and mailing lists, while informal platforms included student-led groups and peer-shared networks. The questionnaire was distributed to students from more than ten universities and over thirty faculties across multiple regions of Saudi Arabia, covering medical, health sciences, and non-medical disciplines across all academic years. Participation was voluntary, anonymous, and unrestricted, allowing respondents to complete the survey at their convenience. No incentives were offered for participation, and measures were implemented to limit duplicate responses where possible. Electronic informed consent was obtained from all participants prior to accessing the questionnaire. Due to the use of social media-based convenience sampling, the response rate could not be determined, and clustering of participants within shared networks may have occurred. As a result, the sample should not be considered nationally representative.

### 2.3 Sample size calculation

The minimum required sample size was calculated based on the expected prevalence of substance use among university students. A Saudi study published in 2024 reported a prevalence of 20.2% for substance use among students in health-related colleges (14). Using this estimate, the sample size was calculated with OpenEpi (Version 3), an open-source epidemiologic sample size calculator, assuming a 95% confidence level. The calculation assumed a precision (margin of error) of 5% and a design effect of 1.0 for simple random sampling. The initial minimum sample size was 248 participants. To account for the non-probability sampling approach and increase the target sample size, a design effect of 3 was applied, resulting in a final required sample size of 744 students, which was achieved.

### 2.4 Study instrument

Data were collected using a structured, self-administered questionnaire adapted from the World Health Organization Alcohol, Smoking, and Substance Involvement Screening Test (ASSIST), version 3.0 (15). The ASSIST v3.0 is a validated screening tool developed by the World Health Organization for detecting and managing substance use and related risks in primary care settings. The ASSIST is available in English and ten other languages, including Arabic. The validated Arabic version of the ASSIST v3.0 was used in this study.

Due to the sensitive nature of substance use and the sociocultural context in Saudi Arabia, only the initial section of the ASSIST was administered. This section assesses lifetime use of a range of psychoactive substances and was selected to minimize respondent discomfort, reduce social desirability bias, and enhance participation and reporting accuracy. As only the lifetime use section was utilized, standard ASSIST risk scores could not be calculated, limiting clinical risk stratification.

The adapted questionnaire assessed lifetime use of tobacco products, alcohol, cannabis, sedatives or sleeping pills, amphetamine-type stimulants, hallucinogens, opioids, cocaine, and inhalants. Participants were asked to report non-medical use of these substances; however, the questionnaire did not distinguish between prescribed and non-prescribed use for sedatives or sleeping pills.

In addition, the questionnaire included items capturing sociodemographic and academic characteristics, as well as psychosocial variables such as self-reported anxiety or depression during the preceding 12 months, peer substance use, and perceived peer pressure to initiate substance use. No formal pilot study was conducted; however, the questionnaire was reviewed by the research team for clarity and content validity prior to dissemination. Internal consistency (e.g., Cronbach’s alpha) was not assessed, as the instrument primarily consisted of standardized and previously validated items.

### 2.5 Statistical analysis

Data were exported, cleaned, and analyzed using R statistical software (version 4.3.3). Data cleaning procedures included checking for duplicate entries, inconsistencies, and incomplete responses. Surveys with substantial missing data were excluded from analysis, and no imputation methods were applied. Descriptive statistics were used to summarize participant characteristics and substance use patterns, including frequencies, percentages, means, and standard deviations. Age was analyzed as a continuous variable in regression analyses and additionally categorized (≤20, 21–23, ≥24 years) for descriptive and bivariate analyses. The distribution of the continuous variable (age) was assessed using appropriate methods (including visual inspection and normality testing), and the assumptions for parametric testing were satisfied prior to applying analysis of variance (ANOVA).

Bivariate analyses were conducted to examine associations between substance use categories and sociodemographic, academic, and psychosocial variables, using analysis of variance (ANOVA) for continuous variables and appropriate tests for categorical variables. Pearson’s chi-square test was used when expected cell counts were adequate, while Fisher’s exact test was applied when expected cell counts were less than 5. Multivariable analyses for non-tobacco substances were explored; however, due to sparse data and instability in model estimates (including non-interpretable confidence intervals), these analyses were not retained. Therefore, tobacco use was selected as the primary outcome for multivariable modeling due to its higher prevalence and suitability for stable estimation. Variables deemed theoretically relevant or statistically significant in bivariate analysis were included in the final model. Adjusted odds ratios (aOR) with corresponding 95% confidence intervals (CI) were reported. Reference categories were explicitly defined for all categorical variables. Multicollinearity among independent variables was assessed using variance inflation factors (VIF), with no evidence of significant collinearity observed. Statistical significance was set at a two-sided *p* value of <0.05.

### 2.6 Minimization of bias

To minimize potential sources of bias, several measures were implemented. To reduce social desirability bias, the questionnaire was anonymous and participation was voluntary. The survey was distributed broadly across multiple universities and academic years to enhance diversity of responses. Non-response bias was minimized by encouraging wide participation and achieving a relatively large sample size across different regions.

## 3 Results

### 3.1 Sample characteristics

A total of 744 university students from universities across all five geographical regions of Saudi Arabia completed the survey. The mean age of the sample was 22.4 years (±2.6), with a slightly higher proportion of males (57%). The vast majority were Saudi nationals (86%) enrolled in public universities (88%), with representation from all five geographical regions. Most participants were from medical or health sciences faculties (71%) and were in their fifth academic year or above (51%). Nearly half (48%) reported a grade point average (GPA) of 4.0 or higher on a 5.0 scale. A quarter of the sample (25%) identified as current cigarette smokers (Table 1).

**Table 1:** Sociodemographic and academic characteristics of participants, Saudi Arabia, November–December 2025.

Variable	Count (%) *n* = 744
Age	22.4 ± 2.6^1^
Sex	
Male	427 (57)
Female	317 (43)
Nationality	
Saudi	643 (86)
Non-Saudi	101 (14)
University type	
Public	654 (88)
Private	90 (12)
Region	
Central	137 (18)
Western	221 (30)
Eastern	80 (11)
Northern	144 (19)
Southern	162 (22)
Faculty	
Medical	526 (71)
Non-medical	218 (29)
Year of study	
1st year	86 (12)
2nd year	83 (11)
3rd year	91 (12)
4th year	105 (14)
5th year or above	379 (51)
GPA	
<3.0	60 (8.1)
3.0–3.99	330 (44)
≥4.0	354 (48)
Current smoker	188 (25)

^1^Mean ± standard deviation (SD); values are presented as n (percentage). GPA, grade point average.

### 3.2 Prevalence of substance use

The lifetime prevalence of any substance use (including tobacco) among participants was 45%. As detailed in Table 2, tobacco products were the most commonly used substance (30.0%). Excluding tobacco, the lifetime prevalence of other substance use was 15%. The most prevalent non-tobacco substances were sedatives or sleeping pills (14.0%) and cannabis (9.7%). Lower prevalence was reported for alcohol (4.8%), amphetamine-type stimulants (e.g., Captagon) (3.4%), hallucinogens (3.6%), and opioids (1.2%). No lifetime use was reported for cocaine or inhalants.

**Table 2:** Lifetime prevalence of substance use among university students, Saudi Arabia, November–December 2025.

Substance type	Count (%) *n* = 744
Have you ever used any of the following substances at any point in your life (for non-medical purposes)?	
Tobacco and tobacco products	
No	521 (70)
Yes	223 (30)
Alcohol	
No	708 (95)
Yes	36 (4.8)
Cannabis (Marijuana, Hashish, Pot)	
No	672 (90)
Yes	72 (9.7)
Cocaine (Coke, crack)	
No	744 (100)
Amphetamine-type stimulants (e.g., Captagon)	
No	719 (97)
Yes	25 (3.4)
Inhalants (e.g., glue, thinners, gasoline)	
No	744 (100)
Sedatives or Sleeping Pills (e.g., Valium, Xanax)	
No	640 (86)
Yes	104 (14)
Hallucinogens (e.g., LSD, acid, mushrooms)	
No	717 (96)
Yes	27 (3.6)
Opioids (e.g., Heroin, Tramadol, Morphine)	
No	735 (99)
Yes	9 (1.2)
Other drugs	
No	691 (93)
Yes	53 (7.1)
Use of substances other than Tobacco (Tobacco excluded)	112 (15)
Substance use overall	335 (45)

Values are presented as number (percentage). Percentages are calculated using the total sample (*n* = 744). Substance use refers to lifetime use unless otherwise specified.

### 3.3 Patterns and determinants: bivariate analysis

For comparative analysis, participants were categorized into three groups: substance-free, tobacco-only users, and users of other substances (with or without tobacco). Table 3 presents significant differences across these groups. Age differed significantly across substance use groups (ANOVA, *p* < 0.001), with tobacco-only users being older on average (mean 23.7 ± 2.45 years) compared with substance-free participants (21.74 ± 2.64 years) and users of other substances (21.85 ± 0.90 years).

**Table 3:** Comparison of participants by substance use status, Saudi Arabia, November–December 2025.

Variable	Substance-free *n* = 409 Count (%)	Tobacco only *n* = 223 Count (%)	Other substances *n* = 112 Count (%)	*p*-value
Age	21.74 ± 2.64	23.72 ± 2.45	21.85 ± 0.90	<0.001^1^
Sex				<0.001^2^
Male	181 (44)	218 (98)	28 (25)	
Female	228 (56)	5 (2.2)	84 (75)	
Faculty				<0.001^2^
Medical	324 (79)	113 (51)	89 (79)	
Non-medical	85 (21)	110 (49)	23 (21)	
GPA				<0.001^2^
<3.0	4 (1.0)	56 (25)	0 (0)	
3.0–3.99	168 (41)	134 (60)	28 (25)	
≥4.0	237 (58)	33 (15)	84 (75)	
Close friends who use drugs	92 (22)	70 (31)	28 (25)	0.049^2^
Have you ever had drugs due to peer pressure	0 (0)	70 (31)	37 (33)	<0.001^3^
Suffered from anxiety or depression in the last 12 months	161 (39)	118 (53)	73 (65)	<0.001^2^

Values are presented as mean ± standard deviation (SD) for continuous variables and number (percentage) for categorical variables. Percentages are calculated within each column. ^1^One-way analysis of variance (ANOVA). ^2^Pearson’s Chi-square test. ^3^Fisher’s exact test. Statistical significance was set at a two-sided *p* value of <0.05. GPA, grade point average.

**Figure 1:**
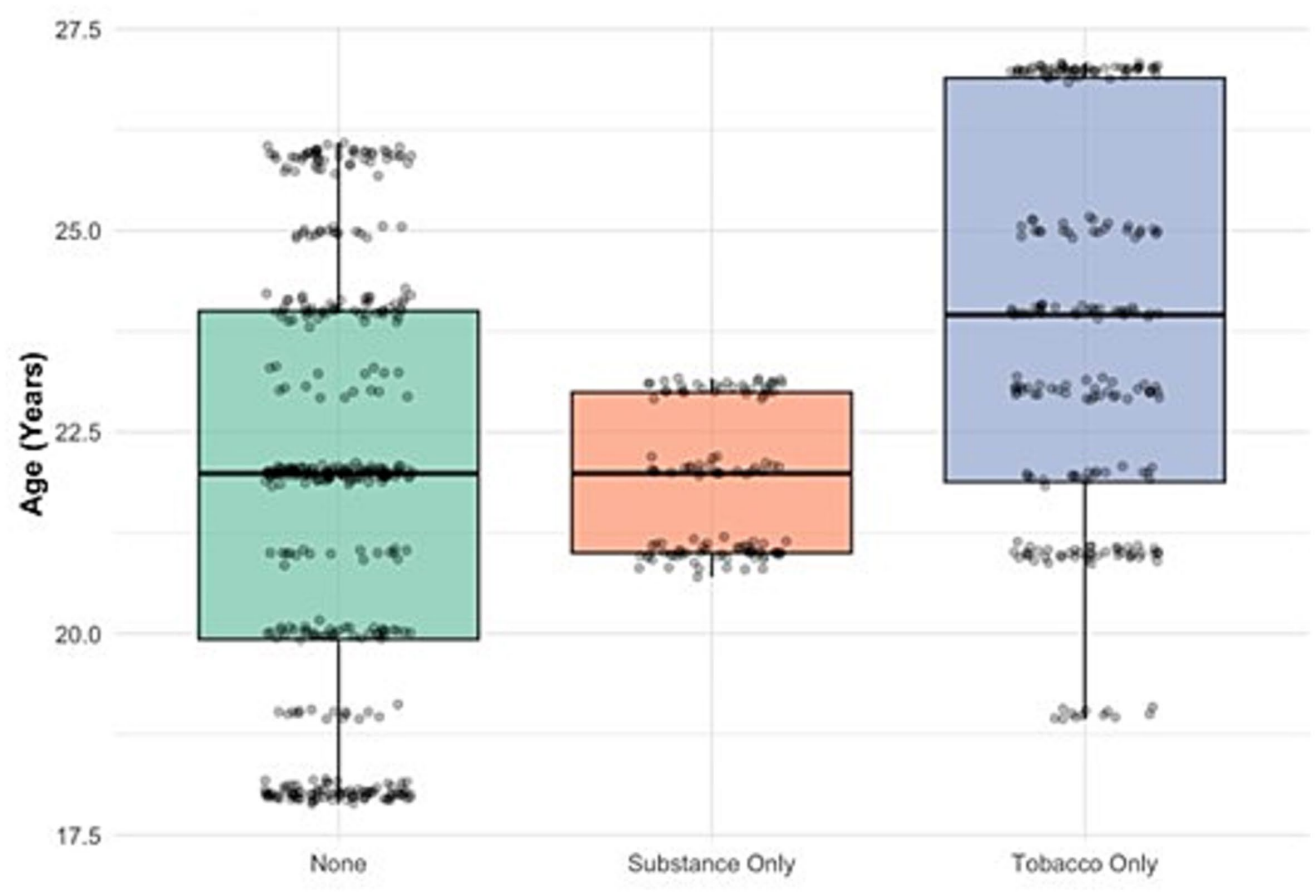
Age distribution across substance use categories among university students in Saudi Arabia, November–December 2025.

Demographically, tobacco-only users were predominantly male (98%) and were the oldest group (mean age 23.7 years). In contrast, among participants reporting non-tobacco substance use, females constituted 75% of users. Figure 1 further illustrates the age distribution across these categories, confirming that the tobacco-only group was consistently older. Academically, a strong inverse relationship was observed between tobacco use and high academic performance. Users of other substances had the highest proportion of high achievers (75% with GPA ≥ 4.0), while tobacco-only users had the lowest (15%). Furthermore, a higher proportion of tobacco-only users were enrolled in non-medical faculties (49%) compared to the other groups.

Regarding psychosocial factors, self-reported anxiety or depression in the past 12 months was most prevalent among users of other substances (65%), followed by tobacco-only users (53%), and was lowest in the substance-free group (39%). The reported presence of close friends who use substances was 31% among tobacco-only users, 25% among users of other substances, and 22% among substance-free participants. All reported instances of substance use due to peer pressure were confined to participants in the substance-using groups, with higher proportions observed among tobacco-only users (31%) and users of other substances (33%).

### 3.4 Gender differences in substance-specific prevalence

**Figure 2:**
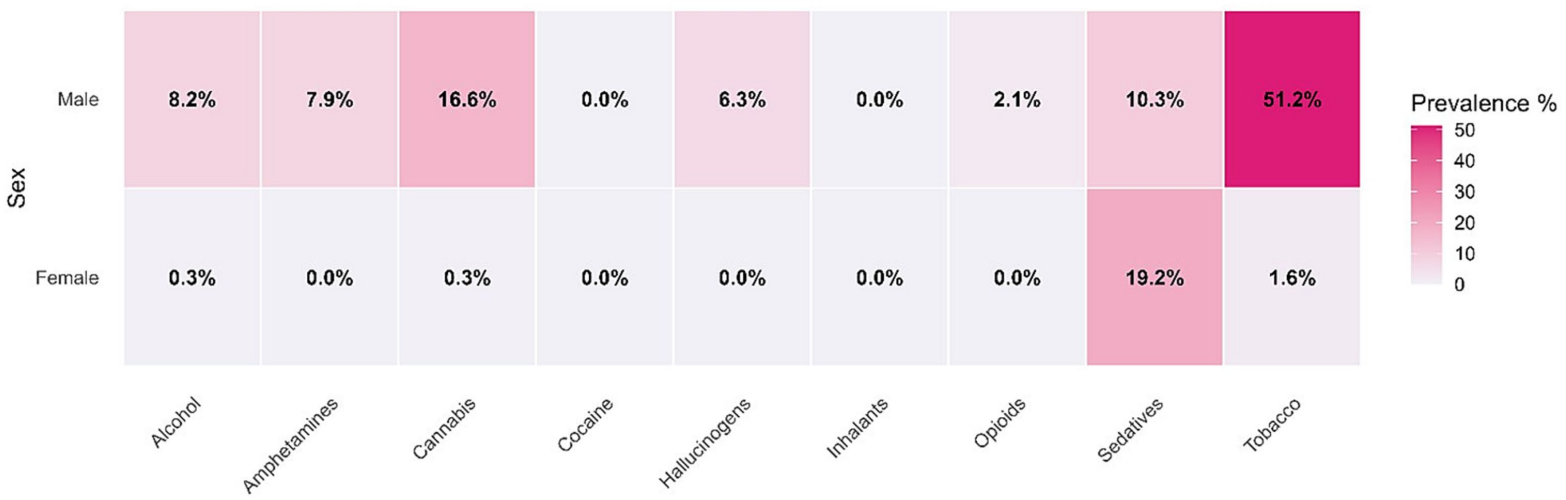
Gender-specific patterns of lifetime substance use among participants with a history of substance use, Saudi Arabia, November–December 2025. Percentages are calculated within participants reporting lifetime substance use.

Figure 2 presents gender-specific patterns in the type of substances used among participants who reported lifetime substance use, rather than the total study population. Percentages are calculated within the subgroup of participants reporting lifetime substance use. Within this subgroup, tobacco was the most commonly reported substance among males, with 51.2% indicating lifetime use, compared with 1.6% of females. Males also showed higher prevalence of cannabis (16.6% vs. 0.3%), alcohol (8.2% vs. 0.3%), hallucinogens (6.3% vs. 0.0%), opioids (2.1% vs. 0.0%), and amphetamine-type stimulants (7.9% vs. 0.0%). In contrast, sedative or sleeping pill use predominated among females, affecting 19.2% compared with 10.3% of males. No lifetime use of cocaine or inhalants was reported in either gender. These findings indicate distinct gender-specific substance use profiles among students with a history of substance use.

### 3.5 Independent determinants of tobacco use

A multivariable logistic regression model identified several independent factors associated with lifetime tobacco use (Table 4). Older age (per one-year increase) was significantly associated with higher odds of tobacco use (aOR = 1.41, 95% CI: 1.26–1.59, *p* < 0.001). Male gender was also strongly associated with increased odds (aOR = 19.8, 95% CI: 8.48–58.0, *p* < 0.001), as was enrollment in a non-medical faculty (aOR = 4.79, 95% CI: 2.73–8.67, *p* < 0.001). Reported peer pressure to use substances remained a significant predictor (aOR = 5.50, 95% CI: 2.24–14.4, *p* < 0.001). In contrast, a GPA of 3.0 or higher was associated with lower odds of tobacco use (aOR = 0.14, 95% CI: 0.04–0.39, *p* < 0.001). Self-reported anxiety or depression was also significantly associated with increased odds of tobacco use (aOR = 3.92, 95% CI: 2.24–7.10, *p* < 0.001). Peer-related variables were evaluated during model development, and variables contributing to model instability were excluded from the final model.

**Table 4:** Multivariable logistic regression for tobacco use among university students, Saudi Arabia, November–December 2025.

Characteristic	Adjusted OR [95% CI]	*p*-value
Age	1.41 [1.26–1.59]	<0.001
Sex		
Female	Reference	
Male	19.8 [8.48–58.0]	<0.001
Faculty		
Medical	Reference	
Non-medical	4.79 [2.73–8.67]	<0.001
GPA		
<3.0	Reference	
≥3.0	0.14 [0.04–0.39]	<0.001
Peer pressure		
No	Reference	
Yes	5.50 [2.24–14.4]	<0.001
Anxiety/depression		
No	Reference	
Yes	3.92 [2.24–7.10]	<0.001

Adjusted odds ratios (aOR) and 95% confidence intervals (CI) were estimated using multivariable logistic regression. Age was modeled as a continuous variable (per one-year increase). Variables included in the model were selected based on theoretical relevance and statistical significance in bivariate analysis. Variables with conceptual overlap were evaluated, and those contributing to model instability were excluded from the final model. Statistical significance was set at a two-sided *p* value of <0.05.

## 4 Discussion

This multi-regional convenience sample provides an overview of substance use patterns among university students in Saudi Arabia, identifying prevalence estimates, substance-specific profiles, and key demographic, academic, and psychosocial correlates. The findings contribute to the growing body of evidence on substance use among young adults in the region and highlight several context-specific patterns that warrant attention from policymakers and university health services. However, these findings should be interpreted cautiously, as the use of lifetime measures does not capture current substance use patterns or severity of involvement. In addition, the convenience sampling approach and overrepresentation of students from medical and health sciences faculties may limit the generalizability of the findings to the broader university student population in Saudi Arabia.

Nearly half of the surveyed students reported lifetime use of at least one substance when tobacco was included, while the prevalence of non-tobacco substance use remained considerably lower. This distinction is critical in the Saudi context, where tobacco use is socially more tolerated and legally accessible compared with other psychoactive substances. Similar to prior studies from neighboring countries, tobacco emerged as the most prevalent substance, reinforcing its role as the dominant form of substance use among university students in conservative settings (16).

Smoking remains a major preventable public health issue with global impacts on morbidity and mortality. In Saudi Arabia, tobacco use is common, especially among adolescents and young adults. The prevalence observed in this study reflects lifetime tobacco use and is therefore not directly comparable to studies reporting current smoking prevalence. This study found a higher prevalence compared with the 11.65% at King Saud University and the 16.2% nationwide reported by Albuhairan et al., as well as the 20.7% in Jazan (17–19). These differences should be interpreted cautiously given variations in measurement approaches. Internationally, prevalence varies: 17.2% in Nairobi, 20.7% in India, and 22.5% in Egypt (20–22). In contrast, countries like Canada report very low rates, around 6.8% (23).

The relatively low prevalence of alcohol and illicit drug use observed in this study contrasts sharply with reports from Western and some African settings, where alcohol predominates among university students (6–8). This discrepancy is likely explained by legal restrictions, religious prohibitions, and cultural norms in Saudi Arabia, which limit availability and increase social and legal consequences of alcohol and illicit drug use (9). Additionally, underreporting may have occurred due to sociocultural stigma and legal sensitivity surrounding substance use. These contextual factors have consistently been shown to shape substance use profiles across regions, rather than eliminating substance use.

Notably, sedatives and sleeping pills constituted the most commonly reported non-tobacco substances. This pattern has been observed in other studies among health-related student populations in Saudi Arabia and may reflect a combination of academic stress, sleep disturbances, and relatively easier access to prescription medications (14). However, as the questionnaire did not distinguish between prescribed and non-prescribed use, these findings should be interpreted with caution and cannot be assumed to represent misuse. Sedative use in this context may reflect a heterogeneous pattern, including both medically prescribed use and potential non-medical use. Although non-tobacco substances, particularly sedatives, were examined in descriptive and bivariate analyses, multivariable modeling was not feasible due to sparse data and instability in regression estimates, and therefore these findings should be interpreted with caution. Notably, 7.1% of participants reported use of “other drugs,” which was higher than the reported use of some specific substances such as hallucinogens and amphetamine-type stimulants. This category likely represents a heterogeneous group of substances not explicitly captured by the survey instrument. In the Saudi context, this may include emerging or less commonly studied substances; however, the lack of specificity limits interpretation and highlights a measurement limitation of the instrument.

Marked gender-specific patterns were identified, with tobacco, hallucinogens, amphetamines, and alcohol use being more prevalent among males, while sedative use was more frequently reported by females. Importantly, users of non-tobacco substances were predominantly female (75%), representing one of the most striking findings in the study and highlighting distinct gender-specific patterns of substance use. These findings align with emerging regional evidence suggesting that gender differences in substance use are substance-specific rather than uniform. While males continue to dominate in socially visible and traditionally stigmatized substances, females may be more likely to engage in the use of prescription or medically framed substances, possibly as a coping strategy for stress, anxiety, or sleep problems (24, 25).

An inverse relationship between tobacco use and academic performance was observed, with tobacco-only users exhibiting the lowest proportion of high academic achievers (26, 27). This finding is consistent with prior research demonstrating that substance use can negatively affect concentration, motivation, attendance, and cognitive functioning. Conversely, students using non-tobacco substances, specifically sedative use, demonstrated higher GPAs, a finding that appears counterintuitive and diverges from much of the existing literature (28). The finding that tobacco-only users were older than other groups may reflect greater cumulative exposure over time, increased social independence, or longer duration of risk behaviors, all of which may contribute to higher likelihood of tobacco use among older students.

This paradoxical association may be explained by several factors. One possible explanation is that some high-achieving students may be more inclined to use certain substances, particularly sedatives or stimulants, as perceived tools for managing academic pressure, sleep deprivation, or performance demands (19, 29). Alternatively, this may reflect differential reporting bias or selective use of substances perceived as “functional” rather than recreational. However, given that the questionnaire did not distinguish between prescribed and non-prescribed use, and the cross-sectional design relies on lifetime measures, these findings should be interpreted with caution. Causal relationships cannot be established.

Consistent with prior studies, substance use was associated with higher self-reported rates of anxiety or depression, particularly among users of non-tobacco substances (30, 31). In the multivariable model, self-reported anxiety or depression was also significantly associated with higher odds of tobacco use, suggesting that psychological distress may play an important role in substance use behaviors. It is plausible that psychological distress contributes to the initiation or continuation of substance use, while substance use itself may exacerbate mental health symptoms, creating a bidirectional relationship (32).

Peer influence emerged as a critical determinant, with peer pressure showing a strong independent association with tobacco use (33). During model development, peer-related variables were carefully evaluated due to potential conceptual overlap. The variable “having friends who use substances” was excluded from the final multivariable model because it introduced instability in effect estimates and reduced interpretability. This likely reflects the fact that different peer-related measures may capture overlapping but distinct dimensions of social influence, such as direct behavioral pressure versus broader social exposure (34). Retaining peer pressure in the final model provided a more stable and theoretically consistent representation of peer influence. These findings highlight the importance of carefully distinguishing between different aspects of peer influence when examining social determinants of substance use.

### 4.1 Implications for prevention and policy

The findings underscore the need for nuanced, context-specific prevention strategies within Saudi universities. Tobacco control remains a priority, particularly among male students and those in non-medical faculties. At the same time, the high prevalence of sedative use, especially among females and high-performing students, calls for improved regulation of prescription medications, enhanced mental health screening, and accessible non-pharmacological stress management interventions.

These findings align with national health priorities, including Saudi Vision 2030, which emphasizes the promotion of preventive healthcare and mental well-being among young populations. Universities should consider integrating substance use screening with academic counseling and mental health services, rather than treating substance use as an isolated issue. Culturally sensitive awareness campaigns and student support programs are essential to address stigma and encourage early help-seeking behaviors. Peer-led interventions may also be particularly effective, given the strong influence of social networks observed in this and other studies. Additionally, the inability to perform stable multivariable analyses for non-tobacco substances limits inference regarding their independent determinants.

### 4.2 Limitations

Several limitations should be acknowledged. The cross-sectional design precludes causal inference, and self-reported data are subject to recall and social desirability bias, particularly in a context where substance use is stigmatized. The use of convenience sampling through social media platforms introduces potential selection bias, limits the ability to determine response rates, and may result in clustering of participants within specific networks or institutions. The overrepresentation of students from medical and health sciences faculties (approximately 71%) limits the generalizability of the findings to the broader university student population. Additionally, the use of only the lifetime section of the ASSIST tool precluded classification of participants into standard risk categories, limiting clinical interpretation of substance use severity. Additionally, the inability to distinguish between prescribed and non-prescribed sedative use limits interpretation of these findings and precludes conclusions regarding misuse. Measurement bias may also be present, as key variables such as GPA and mental health status were self-reported. Moreover, Self-reported data may be subject to desirability bias, which may lead to overestimation or underreporting of substance use behaviors, particularly in a health-professional trainee population where awareness and stigma may influence reporting. Despite these limitations, the large sample size and inclusion of students from multiple regions provide valuable insights into substance use patterns within a multi-regional sample of university students in Saudi Arabia.

## 5 Conclusion

Substance use among a multi-regional sample of university students in Saudi Arabia demonstrates distinct and important patterns. Tobacco was the most prevalent substance (30%), while sedatives and sleeping pills emerged as the most common non-tobacco substances (14%), raising concerns given the inability to distinguish prescribed from non-prescribed use. Notably, non-tobacco substance use was predominantly reported by females (75%), highlighting clear gender-specific patterns. In contrast, tobacco use was strongly associated with male gender, older age, and non-medical faculty enrollment, while higher academic performance was associated with lower odds of tobacco use. An unexpected finding was the higher academic performance observed among users of non-tobacco substances, which warrants further investigation. These findings should be interpreted in light of the study’s cross-sectional design and reliance on lifetime measures, which limit causal inference and clinical risk stratification. Addressing these patterns requires integrated, culturally sensitive interventions that combine academic support, mental health services, and targeted substance use prevention. Strengthening university-based prevention programs, enhancing mental health support services, and promoting awareness through culturally appropriate strategies are essential steps toward reducing substance use among students. Future research using longitudinal and multi-center designs is recommended to better understand temporal relationships and evolving substance use patterns. These findings inform evidence-based decision-making in higher education and public health systems to reduce substance use among university students.

## Data Availability

The raw data supporting the conclusions of this article will be made available by the authors, without undue reservation.
